# Supplementary material for: Single Cell Transcriptomic and Chromatin Profiles Suggest Layer Vb Is the Only Layer With Shared Excitatory Cell Types in the Medial and Lateral Entorhinal Cortex
Source: Front Neural Circuits. 2022 Jan 26;15:806154. doi: 10.3389/fncir.2021.806154 (PMC8826650; doi:10.3389/fncir.2021.806154)
Supplement: Supplementary file 14 [file Data_Sheet_1.ZIP › Deep_vs_Sup_Motifs/homerResults/motif23.similar.html]

motif23

## Information for motif23

C
T
A
G
C
G
A
T
T
C
A
G
G
A
C
T
T
C
A
G
A
G
C
T
C
T
A
G
G
C
A
T
  
Reverse Opposite:  

C
G
T
A
A
G
T
C
T
C
G
A
A
G
T
C
C
T
G
A
A
G
T
C
G
C
T
A
G
A
T
C
  

|  |  |
| --- | --- |
| p-value: | 1e-9 |
| log p-value: | -2.190e+01 |
| Information Content per bp: | 1.653 |
| Number of Target Sequences with motif | 5134.0 |
| Percentage of Target Sequences with motif | 55.22% |
| Number of Background Sequences with motif | 16940.8 |
| Percentage of Background Sequences with motif | 52.01% |
| Average Position of motif in Targets | 413.0 +/- 277.1bp |
| Average Position of motif in Background | 375.1 +/- 243.7bp |
| Strand Bias (log2 ratio + to - strand density) | 0.2 |
| Multiplicity (# of sites on avg that occur together) | 1.80 |
| Motif File: | file (matrix) reverse opposite |

### Similar de novo motifs found

|  |  |  |  |  |  |  |  |
| --- | --- | --- | --- | --- | --- | --- | --- |
| Rank | Match Score | Redundant Motif | P-value | log P-value | % of Targets | % of Background | Motif file |
| 1 | 0.838 | C T A G G A C T T A C G A G C T T A C G A G C T T A C G G A C T A T C G A G C T | 1e-6 | -14.075155 | 9.54% | 8.14% | motif file (matrix) |
| 2 | 0.713 | C G A T T A C G C G A T T C A G G A C T T A C G A G C T T A C G A G C T T A C G A C G T T A C G | 1e-6 | -13.911328 | 9.58% | 8.19% | motif file (matrix) |
